# The prevention of progression of arterial disease and diabetes (POPADAD) trial: factorial randomised placebo controlled trial of aspirin and antioxidants in patients with diabetes and asymptomatic peripheral arterial disease

**DOI:** 10.1136/bmj.a1840

**Published:** 2008-10-16

**Authors:** Jill Belch, Angus MacCuish, Iain Campbell, Stuart Cobbe, Roy Taylor, Robin Prescott, Robert Lee, Jean Bancroft, Shirley MacEwan, James Shepherd, Peter Macfarlane, Andrew Morris, Roland Jung, Christopher Kelly, Alan Connacher, Norman Peden, Andrew Jamieson, David Matthews, Graeme Leese, John McKnight, Iain O’Brien, Colin Semple, John Petrie, Derek Gordon, Stuart Pringle, Ron MacWalter, Prevention of Progression of Arterial Disease and Diabetes Study Group, Diabetes Registry Group, and Royal College of Physicians Edinburgh

**Affiliations:** 1 Institute of Cardiovascular Research, University of Dundee, Ninewells Hospital, Dundee DD1 9SY; 2 Diabetes Centre, Glasgow Royal Infirmary; 3 Department of Medicine, Royal Victoria Hospital, Kirkcaldy; 4 British Heart Foundation Glasgow Cardiovascular Research Centre, University of Glasgow; 5 Royal Victoria Infirmary, Newcastle upon Tyne; 6 Department of Biochemistry, Glasgow Royal Infirmary; 7 Department of Medical Cardiology, Glasgow Royal Infirmary; 8 Medical Statistics Unit, University of Edinburgh; 9 Diabetes Research Centre, Ninewells Hospital; 10 Diabetes and Endocrinology, Ninewells Hospital; 11 Diabetes Centre, Stirling Royal Infirmary; 12 Diabetes Centre, Perth Royal Infirmary; 13 Diabetes Centre, Falkirk and District Royal Infirmary, Falkirk; 14 Diabetes Centre, Queen Margaret Hospital, Dunfermline; 15 Diabetes Centre, Monklands Hospital, Airdrie; 16 Metabolic Unit, Western General Hospital, Edinburgh; 17 Wishaw General Hospital, Wishaw; 18 Southern General Hospital NHS Trust, Glasgow; 19 Medical Unit B, Stobhill NHS Trust, Glasgow; 20 Cardiology Department, Ninewells Hospital

## Abstract

**Objective** To determine whether aspirin and antioxidant therapy, combined or alone, are more effective than placebo in reducing the development of cardiovascular events in patients with diabetes mellitus and asymptomatic peripheral arterial disease.

**Design** Multicentre, randomised, double blind, 2×2 factorial, placebo controlled trial.

**Setting** 16 hospital centres in Scotland, supported by 188 primary care groups.

**Participants** 1276 adults aged 40 or more with type 1 or type 2 diabetes and an ankle brachial pressure index of 0.99 or less but no symptomatic cardiovascular disease.

**Interventions** Daily, 100 mg aspirin tablet plus antioxidant capsule (n=320), aspirin tablet plus placebo capsule (n=318), placebo tablet plus antioxidant capsule (n=320), or placebo tablet plus placebo capsule (n=318).

**Main outcome measures** Two hierarchical composite primary end points of death from coronary heart disease or stroke, non-fatal myocardial infarction or stroke, or amputation above the ankle for critical limb ischaemia; and death from coronary heart disease or stroke.

**Results** No evidence was found of any interaction between aspirin and antioxidant. Overall, 116 of 638 primary events occurred in the aspirin groups compared with 117 of 638 in the no aspirin groups (18.2% *v* 18.3%): hazard ratio 0.98 (95% confidence interval 0.76 to 1.26). Forty three deaths from coronary heart disease or stroke occurred in the aspirin groups compared with 35 in the no aspirin groups (6.7% *v *5.5%): 1.23 (0.79 to 1.93). Among the antioxidant groups 117 of 640 (18.3%) primary events occurred compared with 116 of 636 (18.2%) in the no antioxidant groups (1.03, 0.79 to 1.33). Forty two (6.6%) deaths from coronary heart disease or stroke occurred in the antioxidant groups compared with 36 (5.7%) in the no antioxidant groups (1.21, 0.78 to 1.89).

**Conclusion** This trial does not provide evidence to support the use of aspirin or antioxidants in primary prevention of cardiovascular events and mortality in the population with diabetes studied.

**Trial registration** Current Controlled Trials ISRCTN53295293.

## Introduction

Cardiovascular disease is the major cause of morbidity and mortality in patients with type 1 or type 2 diabetes mellitus. These patients have been reported to have an overall mortality of 50% within 40 years of diagnosis compared with only 10% in a control population.^1^ This increased mortality is mainly from ischaemic heart disease^2^ ^3^ and cerebrovascular disease, the incidence of which is reported to be twofold to fivefold greater^4^ ^5^ than the general population without diabetes.

Peripheral arterial disease is another powerful indicator of systemic atheroma. Regardless of whether symptoms are evident,^6^ patients with peripheral arterial disease have an increased risk of subsequent myocardial infarction and stroke and are six times more likely to die from cardiovascular disease within 10 years than patients without peripheral arterial disease.^7^ Patients with peripheral arterial disease have a 15 year accrued survival rate of about 22% compared with a survival rate of 78% in patients without such disease.^8^ ^9^

The use of antiplatelet agents is known to reduce future secondary cardiovascular events in patients with both diabetes mellitus and cardiovascular disease^4^ ^5^ and in patients with peripheral arterial disease.^4^ ^10^ In the population with peripheral arterial disease the results were driven mainly by non-aspirin antiplatelet drugs, although it is often, and incorrectly, assumed that the antiplatelet drug studied in peripheral arterial disease was aspirin. The strength of the evidence for use of antiplatelet agents as secondary prevention in these groups^11^ ^12^ ^13^ ^14^ has, however, led to the suggestion that aspirin might be useful for primary prevention in both diabetes and asymptomatic peripheral arterial disease. These recommendations have been incorporated into international society guidelines such as the joint societies guidelines,^15^ and national guidelines such as the prevention guideline on coronary artery disease from the Scottish Intercollegiate Guidelines Network,^16^ the American Heart Association,^17^ the American Diabetes Association,^18^ and the American College of Cardiology guidelines on peripheral arterial disease.^19^ These guidelines are published despite the evidence from the antithrombotic trialist meta-analysis^4^ that showed no benefit from antiplatelet therapy for primary prevention in people with diabetes. Few data are available on aspirin for primary prevention in patients with diabetes. The primary prevention project trial^20^ compared aspirin with placebo in patients with type 2 diabetes without established cardiovascular disease. Aspirin failed to achieve a significant difference in the composite cardiovascular end point. Evidence is also conflicting on the effects of aspirin on stroke outcomes in patients with diabetes.^21^ Despite the guidelines’ recommendations, antiplatelet uptake as a primary prevention in diabetes has been low, reflecting the uncertainty of the value of this therapy, combined with the knowledge of the significant side effects that can be associated with aspirin usage.^22^ ^23^

A meta-analysis^24^ of four randomised controlled trials of aspirin as primary prophylaxis against cardiovascular events showed that although aspirin decreased the risk of myocardial infarction it did not reduce total mortality and might increase the risk of stroke and of major bleeding. That meta-analysis and another study^25^ concluded that on the basis of evidence from randomised controlled trials aspirin should not be given to all people with diabetes for primary prophylaxis of cardiovascular events but only to specific subgroups.

Several haemostatic and fibrinolytic abnormalities have been detected in patients with diabetes but the most compelling and reproducible abnormalities have been those of platelet behaviour, with enhanced release of platelet products and platelet aggregation^26^; thus the suggestion that aspirin and other antiplatelet agents may be beneficial. Links between platelet aggregation and the increase in oxidative stress seen in people with diabetes^27^ and in those with peripheral arterial disease^28^ have been studied. Free radicals have been shown to increase platelet aggregation, with antioxidants decreasing aggregation.^29^ Defence against free radical attack is provided in part by the body’s antioxidants. Plasma vitamin E and ascorbic acid levels are lowered in people with diabetes. Other scavengers have also been reported to be decreased in people with diabetes and in those with both diabetes and peripheral arterial disease compared with those with peripheral arterial disease but no diabetes.^28^ For these reasons interest has arisen in assessing antioxidant therapy in people with diabetes.^27^ We determined whether aspirin and antioxidant therapy, combined or alone, are more effective than placebo in reducing the development of cardiovascular events in patients with diabetes and asymptomatic peripheral arterial disease.

## Methods

The prevention of progression of arterial disease and diabetes (POPADAD) trial was a multicentre, randomised, double blind, placebo controlled trial. We used a 2×2 factorial design^30^ to examine the efficacy and safety of aspirin plus antioxidant compared with aspirin alone, antioxidant alone, and placebo.

The interventions were daily aspirin 100 mg or placebo tablet, plus antioxidant or placebo capsule. The antioxidant capsule contained α-tocopherol 200 mg, ascorbic acid 100 mg, pyridoxine hydrochloride 25 mg, zinc sulphate 10 mg, nicotinamide 10 mg, lecithin 9.4 mg, and sodium selenite 0.8 mg. We selected this particular mix following advice from experts in antioxidants, with approval of the Medicines’ Control Agency for use as a nutritional aid in clinical studies. The placebo tablet and capsule were identical in appearance to the active tablet and capsule.

### Participants

Participants were recruited by the Royal College of Physicians, Edinburgh, East of Scotland Diabetic Registry Group, which covers diabetic clinics in hospitals in the south east of Scotland and in central Scotland, and from clinics in the west of Scotland. The coordinating centre was Ninewells Hospital, Dundee. Sixteen hospital centres participated in the trial, supported by 188 primary care groups.

Inclusion criteria were adults of either sex, aged 40 or more, with type 1 or type 2 diabetes who were determined as having asymptomatic peripheral arterial disease as detected by a lower than normal ankle brachial pressure index (≤0.99). We selected a higher cut-off point (0.99 *v* 0.9) for the trial as it is recognised that calcification in the vessels of people with diabetes can produce normal or high ankle brachial pressure indexes, even in the presence of arterial disease. We excluded people with evidence of symptomatic cardiovascular disease; those who use aspirin or antioxidant therapy on a regular basis; those with peptic ulceration, severe dyspepsia, a bleeding disorder, or intolerance to aspirin; those with suspected serious physical illness (such as cancer), which might have been expected to curtail life expectancy; those with psychiatric illness (reported by their general practitioner); those with congenital heart disease; and those unable to give informed consent.

### Trial procedures

Potentially eligible participants attending the diabetes clinics during the enrolment period were invited to join the trial. If they agreed they were screened for eligibility. We scrutinised each patient’s hospital and general practice records for evidence of symptomatic cardiovascular disease. We excluded those not currently free from symptoms of vascular disease at the time of this screening visit. Ankle brachial pressure was measured in asymptomatic patients using a standardised technique.^31^ Training was provided to nurses without experience of measuring this pressure. Patients with an ankle brachial pressure index of less than 1.00 were eligible for inclusion.

After providing written informed consent, patients were randomly assigned to one of four treatment groups: aspirin plus antioxidant, aspirin plus placebo, antioxidant plus placebo, or double placebo. The allocation sequence used randomised permuted blocks of eight and was computer generated by the trial statisticians. To ensure allocation concealment an independent pharmacist packaged the drugs into numbered containers. Recruiting nurses dispensed the trial drugs on the day of randomisation, under the authority of the consultant or primary care doctor. Participants also received standard therapy as appropriate (for example, statins, β blockers) at the discretion of the investigator and other responsible clinicians. We emphasised to the investigators the use of appropriate background cardiovascular risk reduction therapy according to current international guidelines. The participants, research nurses, and staff involved in providing care were blinded to group assignment.

Follow-up evaluations were done every six months. At these visits we recorded outcome events, adverse events, and interventions. The results of electrocardiography were recorded at the baseline visit and annually thereafter. The electrocardiograms were reviewed manually for evidence of silent myocardial infarction on the basis of criteria from the Minnesota code. When relevant, a copy of data from hospital admissions was obtained for use by the committee deciding on the presence or absence of a specific end point using predefined criteria. For example, any electrocardiograms obtained from a hospital admission in the case of a suspected myocardial infarction were also coded using the criteria of the Minnesota code so that the appearances of the electrocardiograms were considered in a uniform manner along with clinical and biochemical data in reaching a decision. All primary and secondary end points were adjudicated on a blinded basis by the committee.

### Outcome measures

We used two hierarchical composite primary end points: death from coronary heart disease or stroke, non-fatal myocardial infarction or stroke, or above ankle amputation for critical limb ischaemia; and death from coronary heart disease or stroke. Definitions for these events were according to the World Health Organization criteria for the diagnosis of coronary events and strokes (fatal and non-fatal).

The main secondary end points were all cause mortality, non-fatal myocardial infarction, and occurrence of other vascular events, including stroke, transient ischaemic attack, coronary or peripheral arterial bypass surgery, coronary or peripheral arterial angioplasty, development of angina, claudication, or critical limb ischaemia.

### Power calculations

The event rate for similar end points in patients with asymptomatic peripheral arterial disease in the Edinburgh artery study^32^ was 4% per annum. The event rate in the literature at the time of the start of the trial for patients with diabetes was between twofold and threefold that of the population without diabetes.^1^ ^2^ ^3^ We originally planned to recruit 1600 participants and follow-up each for four years. If only one treatment was effective this would provide 90% power to detect a 25% relative reduction in a four year event rate of 28% (8% per annum) as significant at the 5% level. This equates to 392 events occurring during the trial. With this sample size but both treatments equally effective, so slightly lower overall event rates, 343 events would be expected in the four years of follow-up. This would still provide greater than 80% power to detect for each treatment the same relative reduction in event rate as significant.

A slower than expected recruitment rate and lower event rates led to ongoing consideration of recruitment and termination dates by the data monitoring and ethics committee, trial steering committee, and funding body. Eventually 1276 patients were recruited and the final power calculations, undertaken in 2003, projected that if follow-up continued until June 2006 then 256 events would be expected to occur during the trial. This would give 73% power to detect a 25% relative reduction in event rate and 89% power to detect a 30% reduction in event rate if only one treatment was effective.

### Statistical analysis

The statistical analysis followed the plan determined at the start of the trial. The primary and secondary end points were measures of survival. Accordingly, we used a Cox proportional hazards model as the primary method of analysis. We assessed the interventions by fitting terms corresponding to aspirin, antioxidants, and the interaction between these treatments. As there was no evidence of interaction we dropped this term, allowing the overall effect of each intervention to be assessed. We assessed the assumption of proportionality of hazards and we used Kaplan-Meier plots for the survival experience by treatment group. Specific adverse events were assessed using logistic regression with terms corresponding to aspirin, antioxidants, and the interaction between these treatments. As we found no evidence of interaction we dropped this term, allowing the overall effect of each intervention to be assessed. All analyses were done on an intention to treat basis, with two tailed tests of significance used throughout.

The trial was designed, planned, and executed by the trial steering committee in collaboration with the UK Medical Research Council. The trial was done in accordance with good clinical practice regulations.^33^ Experienced research nurses collected the data, which were entered and analysed by the Medical Statistics Unit, University of Edinburgh. The progress of the study was monitored throughout by the data monitoring and ethics committee. This committee met at six monthly or yearly intervals depending on the stage of the trial. No formal stopping rules were used. The principle employed was that early termination for efficacy or futility would only occur if the evidence was assessed to be strong enough to influence practice.

## Results

Adults aged 40 or more with diabetes were screened between November 1997 and July 2001. A total of 1670 patients were potentially eligible and had an ankle brachial pressure index of 0.99 or less. Of these, 1276 (76.4%) gave written informed consent and were randomised; 320 to receive aspirin tablets plus antioxidant capsules, 318 to receive aspirin tablets plus placebo capsules, 320 to receive placebo tablets plus antioxidant capsules, and 318 to receive placebo tablets plus placebo capsules. The treatment groups were similar for baseline characteristics (table 1).

**Table 1 tbl1:** Baseline characteristics. Values are medians (interquartile ranges) unless stated otherwise

Characteristics	Aspirin plus antioxidant (n=320)	Aspirin plus placebo (n=318)	Placebo plus antioxidant (n=320)	Placebo plus placebo (n=318)
Mean (SD) age (years)	61.0 (10.0)	60.0 (10.1)	60.0 (10.3)	60.1 (9.7)
No (%) women	169 (53)	183 (58)	181 (57)	180 (57)
Time since diagnosis of diabetes (years)	6.7 (2.9-12.9)	6.0 (2.7-13.0)	5.7 (2.4-11.7)	6.4 (2.6-11.6)
No (%) treated with insulin	107 (33)	112 (35)	96 (30)	91 (29)
Smoking status:				
No (%) current smokers	105 (33)	99 (31)	106 (33)	87 (27)
No (%) former smokers	113 (35)	107 (34)	111 (35)	116 (36)
No (%) never smokers	102 (32)	112 (35)	103 (32)	115 (36)
Body mass index (kg/m^2^)	29.7 (26.2-33.3)	28.7 (25.2-33.0)	29.4 (26.1-33.5)	29.2 (25.8-33.2)
Mean (SD) systolic blood pressure (mm Hg)	146 (22)	143 (21)	144 (20)	147 (21)
Mean (SD) diastolic blood pressure (mm Hg)	79 (10)	78 (10)	79 (10)	80 (11)
Ankle brachial pressure index	0.90 (0.82-0.95)	0.91 (0.84-0.95)	0.89 (0.81-0.94)	0.90 (0.83-0.96)
Mean (SD) HbA_1c_ level (%)	8.0 (1.8)	8.0 (1.7)	7.9 (1.8)	7.9 (1.7)
Total cholesterol level (mmol/l)	5.5 (4.8-6.2)	5.6 (4.9-6.2)	5.5 (4.9-6.3)	5.5 (4.9-6.2)
Triglyceride level (mmol/l)	2.2 (1.5-3.2)	2.2 (1.5-3.3)	2.3 (1.4-3.4)	2.1 (1.5-3.3)
High density lipoprotein level (mmol/l)	1.2 (1.0-1.5)	1.3 (1.0-1.5)	1.2 (1.0-1.5)	1.2 (1.0-1.5)
Low density lipoprotein level (mmol/l)	3.1 (2.5-3.7)	3.1 (2.5-3.7)	3.2 (2.6-3.9)	3.1 (2.6-3.7)

### Follow-up

Figure 1 shows the flow of participants in the trial. Overall, 1074 participants had their final follow-up in 2006, six had moved away and were lost to follow-up, one withdrew consent after four years, and 195 died during the trial. The median length of follow-up for randomised participants was 6.7 years and for those with a final follow-up in 2006 follow-up ranged from 4.5 to 8.6 years. A total of 8127 patient years of follow-up were completed.

**Figure fig1:**
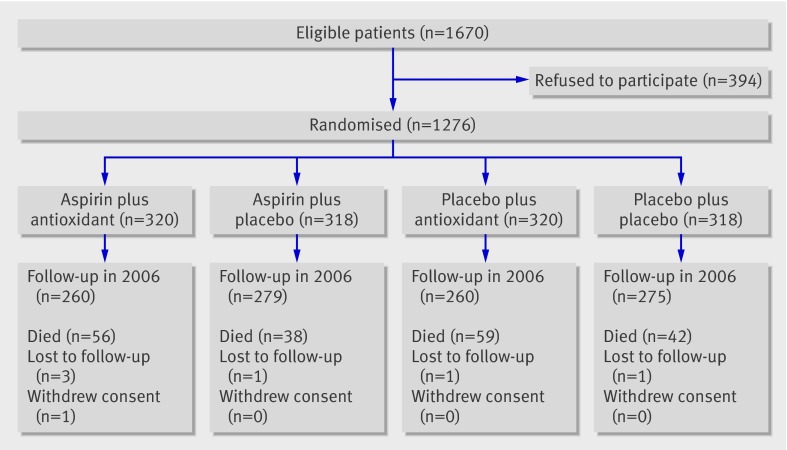
**Fig 1** Progress of participants in trial

### End points

Overall, 233 participants experienced the composite primary end point of death from coronary heart disease or stroke, non-fatal myocardial infarction or stroke, or above ankle amputation for critical limb ischaemia, an overall event rate of 2.9 per 100 patient years. Seventy eight participants died from coronary heart disease or stroke, an event rate of 1.0 per 100 patient years. Table 2 shows the proportion of participants by treatment group who experienced each end point or specific adverse event.

**Table 2 tbl2:** Numbers (percentages) of patients with diabetes who experienced primary end points, secondary end points, and specific adverse events

Variable	Aspirin plus antioxidant (n=320)	Aspirin plus placebo (n=318)	Placebo plus antioxidant (n=320)	Placebo plus placebo (n=318)	P value (interaction*)
Primary end points:					
Composite end point†	58 (18)	58 (18)	59 (18)	57 (18)	0.92
Death from coronary heart disease or stroke	23 (7)	20 (6)	19 (6)	16 (5)	0.90
Secondary end points:					
Death (any cause)	56 (18)	38 (12)	59 (18)	42 (13)	0.93
Death from coronary heart disease	15 (5)	20 (6)	15 (5)	11 (4)	0.24
Stroke death	8 (3)	0 (0)	4 (1)	5 (2)	0.004
Non-fatal myocardial infarction	21 (7)	34 (11)	28 (9)	28 (9)	0.20
Non-fatal stroke	18 (6)	11 (4)	18 (6)	23 (7)	0.14
Above ankle amputation for critical limb ischaemia	6 (2)	5 (2)	4 (1)	5 (2)	0.69
Transient ischaemic attack	3 (1)	11 (4)	8 (3)	12 (4)	0.25
Coronary artery bypass surgery	4 (1)	6 (2)	8 (3)	8 (3)	0.61
Coronary artery angioplasty	3 (1)	4 (1)	4 (1)	4 (1)	0.78
Development of angina	31 (10)	39 (12)	38 (12)	40 (13)	0.59
Peripheral arterial bypass surgery	3 (1)	4 (1)	1 (0.3)	4 (1)	0.44
Peripheral arterial angioplasty	6 (2)	5 (2)	6 (2)	7 (2)	0.71
Development of critical limb ischaemia	13 (4)	8 (3)	9 (3)	10 (3)	0.37
Development of claudication	39 (12)	58 (18)	58 (18)	49 (15)	0.032
Adverse events:					
Malignancy	29 (9)	24 (8)	36 (11)	32 (10)	0.85
Gastrointestinal bleeding	15 (5)	13 (4)	13 (4)	18 (6)	0.36
Gastrointestinal symptoms including dyspepsia	33 (10)	40 (13)	36 (11)	58 (18)	0.31
Arrhythmia	28 (9)	27 (9)	22 (7)	25 (8)	0.67
Allergy, including skin rash	34 (11)	38 (12)	34 (11)	30 (9)	0.47

*Test for interaction between aspirin and antioxidant.

†Death from coronary heart disease or stroke, non-fatal myocardial infarction or stroke, or above ankle amputation for critical limb ischaemia.

The interaction between the aspirin and antioxidant treatments was not statistically significant either for the composite primary end point (P=0.88) or for death from coronary heart disease or stroke (P=0.95). In addition, the interaction between the two treatments was statistically significant for only two of the secondary end points—death from stroke (P=0.004) and the development of claudication (P=0.032). No evidence was found of an interaction for the specific adverse events. Because there was no evidence of an interaction between aspirin and antioxidant, patients in the two groups randomised to receive aspirin were compared with those in the two groups randomised to receive placebo tablets (no aspirin), and patients in the two groups randomised to receive antioxidant were compared with those in the two groups randomised to receive placebo capsules (no antioxidant).

### Aspirin versus no aspirin

Table 3 shows the proportion of participants with each of the primary and secondary end points in the aspirin and no aspirin groups. Figure 2 shows the cumulative percentages of patients over time who experienced each of the primary end points. The differences between these two groups were not statistically significant for either of the primary end points.

**Figure fig2:**
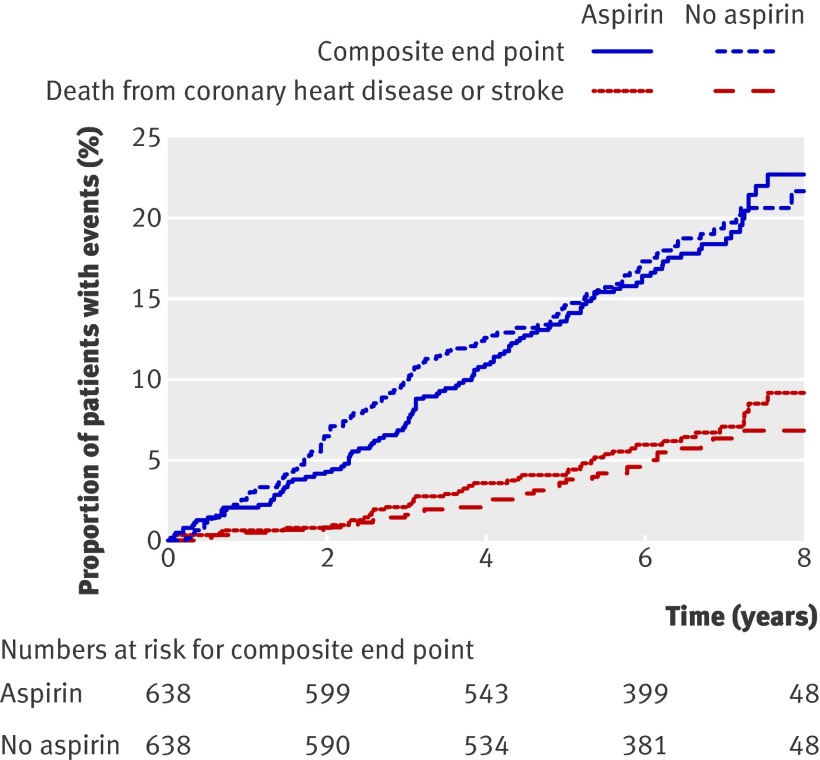
**Fig 2** Kaplan-Meier estimates in aspirin and no aspirin groups of proportion of patients who experienced the composite end point of death from coronary heart disease or stroke, non-fatal myocardial infarction or stroke, or above ankle amputation for critical limb ischaemia; and death from coronary heart disease or stroke

**Table 3 tbl3:** Comparison between aspirin and no aspirin groups in number (percentage) of patients with diabetes who experienced primary end points, secondary end points, and specific adverse events

Variables	Aspirin (n=638)	No aspirin (n=638)	Effect estimate* (95% CI)	P value
Primary end points:				
Composite end point†	116 (18.2)	117 (18.3)	0.98 (0.76 to 1.26)	0.86
Death from coronary heart disease or stroke	43 (6.7)	35 (5.5)	1.23 (0.79 to 1.93)	0.36
Secondary end points:				
Death (any cause)	94 (14.7)	101 (15.8)	0.93 (0.71 to 1.24)	0.63
Coronary heart disease death	35 (5.5)	26 (4.1)	1.35 (0.81 to 2.25)	0.24
Stroke death	8 (1.3)	9 (1.4)	0.89 (0.34 to 2.30)	0.80
Non-fatal myocardial infarction	55 (8.6)	56 (8.8)	0.98 (0.68 to 1.43)	0.93
Non-fatal stroke	29 (4.6)	41 (6.4)	0.71 (0.44 to 1.14)	0.15
Above ankle amputation for critical limb ischaemia	11 (1.7)	9 (1.4)	1.23 (0.51 to 2.97)	0.64
Transient ischaemic attack	14 (2.2)	20 (3.1)	0.70 (0.36 to 1.39)	0.31
Coronary artery bypass surgery	10 (1.6)	16 (2.5)	0.62 (0.28 to 1.38)	0.24
Coronary artery angioplasty	7 (1.1)	8 (1.3)	0.88 (0.32 to 2.43)	0.81
Development of angina	70 (11.0)	78 (12.2)	0.90 (0.66 to 1.25)	0.54
Peripheral arterial bypass surgery	7 (1.1)	5 (0.8)	1.41 (0.45 to 4.43)	0.56
Peripheral arterial angioplasty	11 (1.7)	13 (2.0)	0.85 (0.38 to 1.89)	0.68
Development of critical limb ischaemia	21 (3.3)	19 (3.0)	1.11 (0.60 to 2.06)	0.75
Development of claudication	97 (15.2)	107 (16.8)	0.89 (0.68 to 1.18)	0.42
Adverse events:				
Malignancy	53 (8.3)	68 (10.7)	0.76 (0.52 to 1.11)	0.15
Gastrointestinal bleeding	28 (4.4)	31 (4.9)	0.90 (0.53 to 1.52)	0.69
Gastrointestinal symptoms, including dyspepsia	73 (11.4)	94 (14.7)	0.77 (0.55 to 1.08)	0.081
Arrhythmia	55 (8.6)	47 (7.4)	1.19 (0.79 to 1.78)	0.41
Allergy including skin rash	72 (11.3)	64 (10.0)	1.14 (0.80 to 1.63)	0.47

*Hazard ratios (aspirin *v* no aspirin) for primary and secondary end points and odds ratios (aspirin *v* no aspirin) for adverse events.

†Death from coronary heart disease or stroke, non-fatal myocardial infarction or stroke, or above ankle amputation for critical limb ischaemia.

Figure 3 presents the cumulative percentages of patients over time who died from any cause, along with the percentage expected for all patients based on age and sex specific mortality rates for Scotland, 2002 (General Registrar Office, Scotland). No statistically significant differences were found between the aspirin and no aspirin groups for any of the secondary end points. Specific adverse event rates were not statistically significantly different between the aspirin and no aspirin groups (table 3).

**Figure fig3:**
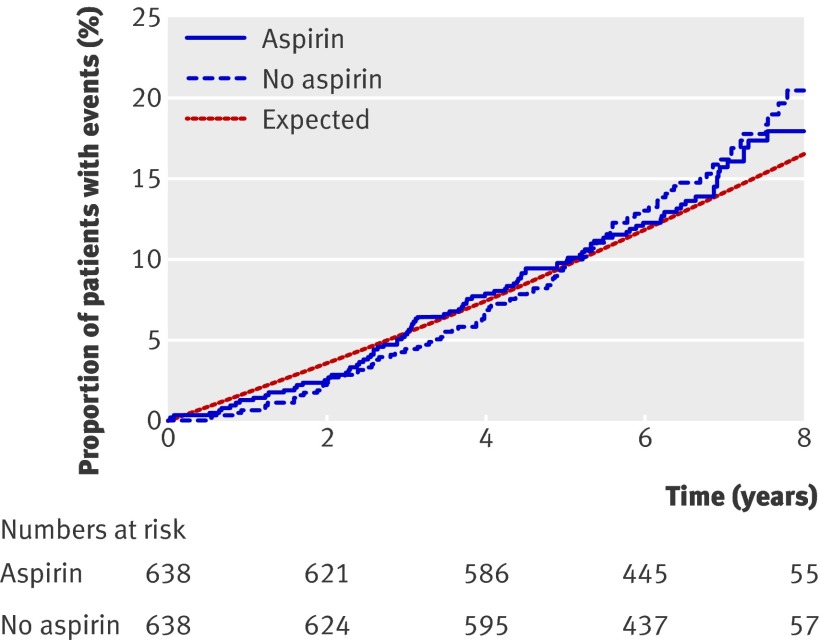
**Fig 3** Kaplan-Meier estimates for aspirin and no aspirin groups of proportion of patients who died from any cause, compared with proportion expected based on age and sex specific population rates for Scotland, 2002

### Antioxidant versus no antioxidant

Table 4 presents the proportion of patients who experienced each of the primary and secondary end points in the antioxidant and no antioxidant groups. Figure 4 shows the cumulative percentages of patients over time who experienced each of the primary end points. No statistically significant differences were found between these two groups for either of the primary end points.

**Figure fig4:**
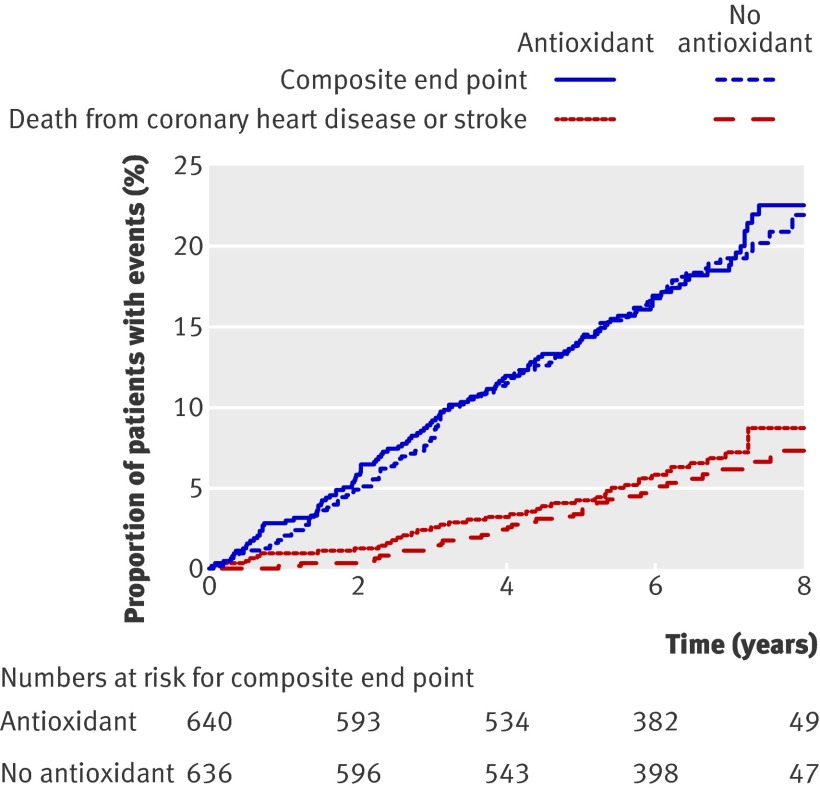
**Fig 4** Kaplan-Meier estimates for antioxidant and no antioxidant groups of proportion of patients who experienced the composite end point of death from coronary heart disease or stroke, non-fatal myocardial infarction or stroke, or above ankle amputation for critical limb ischaemia; and death from coronary heart disease or stroke

**Table 4 tbl4:** Comparison between antioxidant and no antioxidant groups in number (percentage) of patients with diabetes who experienced primary end points, secondary end points, and specific adverse events

Variables	Antioxidant (n=640)	No antioxidant (n=636)	Effect estimate* (95% CI)	P value
Primary end points:				
Composite end point†	117 (18.3)	116 (18.2)	1.03 (0.79 to 1.33)	0.85
Death from coronary heart disease or stroke	42 (6.6)	36 (5.7)	1.21 (0.78 to 1.89)	0.40
Secondary end points:				
Death (any cause)	115 (18.0)	80 (12.6)	1.49 (1.12 to 1.99)	0.006
Coronary heart disease death	30 (4.7)	31 (4.9)	1.01 (0.61 to 1.66)	0.99
Stroke death	12 (1.9)	5 (0.8)	2.49 (0.88 to 7.06)	0.087
Non-fatal myocardial infarction	49 (7.7)	62 (9.7)	0.81 (0.55 to 1.17)	0.26
Non-fatal stroke	36 (5.6)	34 (5.4)	1.08 (0.68 to 1.73)	0.74
Above ankle amputation for critical limb ischaemia	10 (1.6)	10 (1.6)	1.04 (0.43 to 2.50)	0.93
Transient ischaemic attack	11 (1.7)	23 (3.6)	0.49 (0.24 to 1.00)	0.050
Coronary artery bypass surgery	12 (1.9)	14 (2.2)	0.89 (0.41 to 1.91)	0.76
Coronary artery angioplasty	7 (1.1)	8 (1.3)	0.89 (0.32 to 2.46)	0.82
Development of angina	69 (10.8)	79 (12.4)	0.89 (0.65 to 1.23)	0.49
Peripheral arterial bypass surgery	4 (0.6)	8 (1.3)	0.52 (0.16 to 1.72)	0.28
Peripheral arterial angioplasty	12 (1.9)	12 (1.9)	1.05 (0.47 to 2.33)	0.92
Development of critical limb ischaemia	22 (3.4)	18 (2.8)	1.28 (0.68 to 2.38)	0.45
Development of claudication	97 (15.2)	107 (16.8)	0.94 (0.72 to 1.24)	0.67
Adverse events:				
Malignancy	65 (10.2)	56 (8.8)	1.17 (0.80 to 1.71)	0.41
Gastrointestinal bleeding	28 (4.4)	31 (4.9)	0.89 (0.53 to 1.51)	0.67
Gastrointestinal symptoms, including dyspepsia	69 (10.8)	98 (15.4)	0.66 (0.48 to 0.92)	0.015
Arrhythmia	50 (7.8)	52 (8.2)	0.95 (0.64 to 1.43)	0.81
Allergy including skin rash	68 (10.6)	68 (10.7)	0.99 (0.70 to 1.42)	0.97

*Hazard ratios (antioxidant *v* no antioxidant) for primary and secondary end points and odds ratios (antioxidant *v* no antioxidant) for adverse events.

†Death from coronary heart disease or stroke, non-fatal myocardial infarction or stroke, or above ankle amputation for critical limb ischaemia.

Figure 5 shows the cumulative percentages of patients over time who died from any cause, along with the percentage expected for all patients based on age and sex specific population rates for Scotland. The increase in numbers of deaths from any cause in the antioxidant group compared with the no antioxidant group was statistically significant (P=0.006). This difference in all cause mortality seems to be partly due to a relative deficiency of deaths in the no antioxidant group compared with an age and sex matched Scottish population and partly due to a relative excess of deaths in the antioxidant group. No statistically significant differences were found between the antioxidant and no antioxidant groups for any of the other secondary end points.

**Figure fig5:**
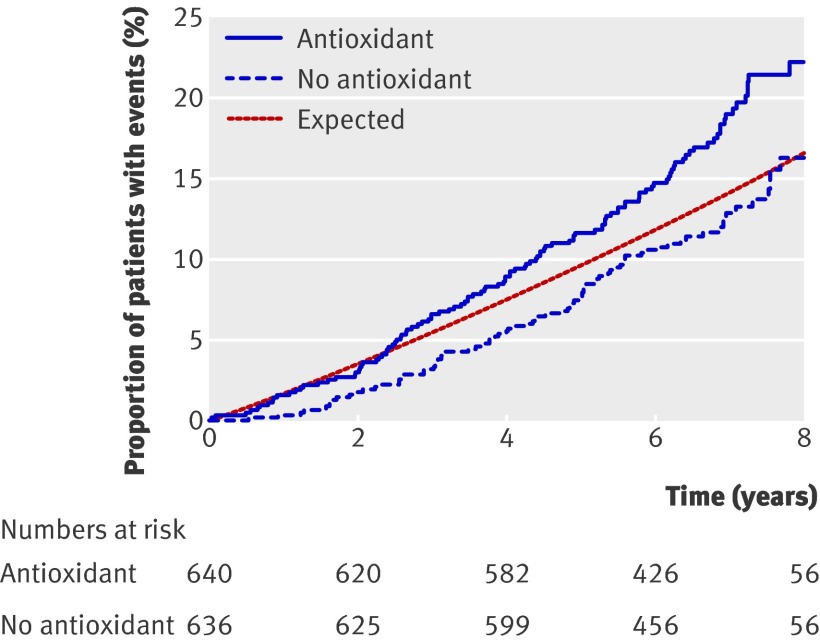
**Fig 5** Kaplan-Meier estimates for antioxidant and no antioxidant groups of proportion of patients who died from any cause, compared with proportion expected based on age and sex specific population rates for Scotland, 2002

Specific adverse event rates were not statistically significantly different in the antioxidant and no antioxidant groups, except for gastrointestinal symptoms including dyspepsia (P=0.015), which were reported by more patients in the no antioxidant groups.

### Cause of death

The end points committee classified the deaths as coronary heart disease, stroke, other cardiac, other vascular, cancer, trauma, or other. The deaths from coronary heart disease were further classified as being due to myocardial infarction or other coronary heart disease, whereas the deaths from stroke were classified as ischaemic, haemorrhagic, or of unknown cause. More deaths occurred in the antioxidant groups than no antioxidant groups for all the categories except other coronary heart disease (table 5).

**Table 5 tbl5:** Cause of death in patients with diabetes according to treatment group

Causes of death	Aspirin plus antioxidant (n=56)	Aspirin plus placebo (n=38)	Placebo plus antioxidant (n=59)	Placebo plus placebo (n=42)
Myocardial infarction	11	10	8	5
Other coronary heart disease	4	10	7	6
Stroke:				
Ischaemic	3	0	3	2
Haemorrhagic	2	0	1	2
Unknown cause	3	0	0	1
Other cardiac	5	0	4	1
Other vascular	3	1	2	2
Cancer	16	9	18	13
Trauma	0	0	2	0
Other	9	8	14	10

### Subgroup analyses

Although subgroup analyses were planned, the trial steering committee asked that analyses of the primary end points compared aspirin and no aspirin groups by subgroups of age, sex, or ankle brachial pressure index. This was because of concern that particular patients with an ankle brachial pressure index of less than 0.90, which defines peripheral arterial disease, may have been at higher risk and so might have shown some benefit in this subgroup. The difference in treatment effect between the subgroups was not statistically significant for any of the three characteristics (table 6).

**Table 6 tbl6:** Comparison of aspirin and no aspirin groups in subgroups of specified baseline characteristics for primary end points

Primary end point	Aspirin			No aspirin		Hazard ratio (95% CI)	P value*
	No of patients	No (%) with event		No of patients	No (%) with event		
**Composite end point†**							
Age (years):							
<60	297	38 (12.8)		315	36 (11.4)	1.11 (0.70 to 1.75)	0.44
≥60	341	78 (22.9)		323	81 (25.1)	0.89 (0.65 to 1.21)	
Women	352	48 (13.6)		361	55 (15.2)	0.89 (0.60 to 1.31)	0.54
Men	286	68 (23.8)		277	62 (22.4)	1.04 (0.74 to 1.47)	
Ankle brachial pressure index:							
≤0.90	314	59 (18.8)		332	75 (22.6)	0.81 (0.58 to 1.14)	0.089
0.91-0.99	324	57 (17.6)		306	42 (13.7)	1.28 (0.86 to 1.91)	
**Death from coronary heart disease or stroke**							
Age (years):							
<60	297	10 (3.4)		315	10 (3.2)	1.07 (0.44 to 2.56)	0.77
≥60	341	33 (9.7)		323	25 (7.7)	1.24 (0.74 to 2.09)	
Women	352	17 (4.8)		361	16 (4.4)	1.09 (0.55 to 2.16)	0.68
Men	286	26 (9.1)		277	19 (6.9)	1.33 (0.73 to 2.40)	
Ankle brachial pressure index:							
≤0.90	314	22 (7.0)		332	24 (7.2)	0.96 (0.54 to 1.71)	0.17
0.91-0.99	324	21 (6.5)		306	11 (3.6)	1.84 (0.89 to 3.82)	

*Test for heterogeneity of treatment effect in subgroups.

†Death from coronary heart disease or stroke, non-fatal myocardial infarction or stroke, or above ankle amputation for critical limb ischaemia.

### Trial drugs

The cumulative percentage of patients who stopped taking the tablets (aspirin or placebo) before they experienced the composite primary end point, died, moved abroad, withdrew consent, or completed the study was 14% by the end of one year. Thereafter withdrawal from trial therapy was fairly constant, leading to a cumulative rate of 50% after five years.

## Discussion

We evaluated the effect of aspirin or antioxidant on cardiovascular events and mortality in a large cohort of people with diabetes mellitus with asymptomatic peripheral arterial disease. These two clinical criteria were selected for study as guidelines^15^ ^16^ ^17^ ^18^ were being published, without evidential support, recommending aspirin use as primary prevention of cardiovascular disease in patients with diabetes mellitus and with asymptomatic peripheral arterial disease. We found no evidence of benefit from either aspirin or antioxidant treatment on the composite hierarchical primary end points of cardiovascular events and cardiovascular mortality. The lower 95% confidence limits for these primary end points, however, only just excluded a 25% benefit for aspirin and a 23% benefit for antioxidant, whereas the upper 95% confidence limits only just excluded a 27% increase in cardiovascular events for aspirin and a 34% increase for antioxidant. Clinically important benefits are unlikely from the results of this study, although it is possible that small effects may be shown with larger trials continued for a longer time.

We preferred the 2×2 factorial design to two separate trials because it had major advantages.^30^ Firstly, it greatly reduced the number of patients needed to achieve the specified statistical power on the assumption that aspirin and antioxidant did not interact. Secondly, using such a design provided an opportunity for testing the interaction between the two interventions, although the power to detect an interaction was low. This design yields double the information of single factorial designs.

In examining why aspirin may have been ineffective the question was asked as to whether these patients were at sufficient risk, in terms of peripheral arterial disease, as the cut-off point of an ankle brachial pressure index of 0.99 or less is higher than that used to define peripheral arterial disease in the population (<0.9).^31^ A subgroup analysis did not, however, find evidence of a difference in effect of aspirin between those with an index of 0.91-0.99 and those below this level. Furthermore, one of the current major interventions in the specialty of diabetes mellitus is statin therapy. Calculations by two of the centres (DM and CK) in over 10 000 people with diabetes showed a mean total cholesterol level of 6.0 mmol/l in 1996 decreasing to 4.3 mmol/l in 2007. As aspirin was the first drug to have an evidence base for secondary prevention of cardiovascular disease it is always given to patients in subsequent trials and it might be asked if aspirin does indeed provide additional benefit when statins are used to good effect.

The importance of the neutral effect of aspirin on cardiovascular events is that this drug is not without side effects.^23^ Aspirin is the most commonly prescribed drug in Scotland, with about 544 438 person years exposure per year in 2002. The number of prescriptions is increasing. The overwhelming majority of this, in the region of Tayside at least, is prescription based, with only about 7% being from over the counter use. Aspirin is one of the top 10 causes of adverse drug events reported to the Commission on Human Medicines. Gastrointestinal bleeding is associated with general use of non-steroidal anti-inflammatory drugs in over 80% of reported cases, and 87% of that use is associated with aspirin, either alone or with other non-steroidal anti-inflammatory drugs.^34^ The risk of a bleeding event increases with age and also continuous exposure.^23^ Although the calculated risk of major bleeding is relatively small,^35^ the number of people taking aspirin is relatively large and therefore in population terms aspirin induced bleeding is a major problem. In a meta-analysis the number needed to treat to cause an adverse event has been calculated as 248,^36^ and this is relevant to the large and increasing population with diabetes.

Of concern was the fact that there was a tendency to harm in the antioxidant group. It should be noted that the increase in number of deaths in the antioxidant groups seems to partly reflect better survival than expected of the groups who did not receive antioxidants, rather than just an obvious negative effect of the antioxidants. Thus this may at least in part be a difference achieved by chance. This agrees with recently published work,^37^ and these data should be added to future systematic reviews and meta-analyses. Anecdotally, many people with diabetes supplement with antioxidants after major publicity in the lay press of a deficiency in antioxidants in such people. We found no evidence for this perceived benefit from our study.

We found no evidence to support the use of either aspirin or antioxidants in the primary prevention of cardiovascular events and mortality in people with diabetes. Aspirin should, however, still be given for secondary prevention of cardiovascular disease in people with diabetes mellitus, when the evidence base is convincing, and the results of this study must not detract from this important standard of care.

**What is already known on this topic**

Aspirin is effective in the secondary prevention of cardiovascular events in patients with symptomatic peripheral arterial disease and with or without diabetes
Aspirin is responsible for significant gastrointestinal morbidity
No large intervention trial has shown any reduction of events with antioxidant intervention

**What this study adds**

Aspirin was not effective in the primary prevention of cardiovascular events in patients with asymptomatic peripheral arterial disease and diabetes
Antioxidants showed no benefit on cardiovascular events in this population

## Notes

We thank Bayer for donating the aspirin and placebo tablets, Scotia Pharmaceuticals (formerly Cardinal) for the anitioxidant capsules and matching placebo, and the study nurse team of the prevention of progression of arterial disease and diabetes trial. Members of the Prevention of Progression of Arterial Disease and Diabetes Study Group. Medical Research Council steering committee: JBel (principal investigator), IC, SC, AMacC (chair), RT, and representatives of the Medical Research Council (various). Data and safety monitoring committee: Keith Fox, Desmond G Johnston, Gordon Murray, and JS (chair). End points committee: PM (chair), SMcE, JMcK, SP, RMacW, JBan, and Mairi Stirling. Contributing centres: Dundee—GL, RJ, AM, and Ray Newton (Ninewells Hospital); Dunfermline—AJ (Queen Margaret Hospital); Edinburgh West—JMcK (Western General Hospital); Falkirk—J Doig and NP (Falkirk and District Royal Infirmary); Glasgow—CS (Southern General Hospital), DG (Stobhill), Colin Kesson (Victoria Infirmary), and JP (Royal Infirmary); East Kilbride—Susan Benbow (Hairmyres and Stonehouse); Kirkcaldy—IC (Royal Victoria Hospital); Livingstone—Stuart Gray (St John’s Hospital); Perth—AC (Perth Royal Infirmary); Monklands—DM (Monklands Hospital); Motherwell and Wishaw—IO’B (Wishaw General); Stirling—Sheila Reith and CK (Stirling Royal Infirmary). Nursing staff: JBan (senior research nurse). Royal College of Physicians Edinburgh Diabetes Registry Group, and West of Scotland clinicians: JBel, IC, John Chalmers, Joycelin Chalmers, AC, John Doig, David Fraser, Stuart Gray, Victor Hawthorne, RJ, RL (study statistician), GL, Susan Lewis, Derreck McCullough, SMcE, Margaret MacLeod, John McKnight, David Matthews, Andrew Morris, Ray Newton, NP, RP (study statistician), Sheila Reit, Mary Scott, Tanya Siann, Alan Smith, and James Walker.

Ethical approval: This work was approved by the local research ethics committees of all participating centres and by a multiresearch ethics committee.

Provenance and peer review: Not commissioned; externally peer reviewed.

## Notes

**Cite this as: ***BMJ* 2008;337:a1840
